# Evaluation of the longer-term impacts on working practices of veterinarians in India after attending a canine surgical neutering training programme

**DOI:** 10.1017/awf.2025.30

**Published:** 2025-05-30

**Authors:** Emma L Rayner, Anahita Kumar, Ilona Airikkala-Otter, Stacy Sequeira, Richard Mellanby, Andrew D Gibson, Luke Gamble, Stella Mazeri

**Affiliations:** 1 Worldwide Veterinary Service, 4 Castle Street, Cranborne, Dorset, UK; 2 Worldwide Veterinary Service, Hicks International Training Centre Madungo Vaddo, near Assagao Panchayat, Assagao, Bardez, Goa, India; 3 Worldwide Veterinary Service, International Training Centre, Gramya Bhavan, RDO trust Building, Aruvankadu, The Nilgiris 643202, Tamil Nadu, India; 4 Royal (Dick) School of Veterinary Studies, The University of Edinburgh, Edinburgh, UK; 5 The Epidemiology, Economics and Risk Assessment (EERA) Group, The Roslin Institute, Royal (Dick) School of Veterinary Studies, Easter Bush, Midlothian, UK

**Keywords:** Animal welfare, continuing professional development, dog welfare, long-term outcomes, practical skills training, veterinary education

## Abstract

Effective, continuing professional development opportunities provide veterinarians with the necessary skills to uphold animal welfare standards. In India, surgical neutering is integral in successfully managing the large, free-roaming dog population; the delivery of skills-based, training opportunities which result in long-term behavioural changes remains challenging. Indian veterinarians attending a 12-day, practically focused, training programme on canine surgical neutering, completed a questionnaire prior to the commencement of training and 10–12 months afterwards. Questions explored the programme’s impact on their attitudes, working practices, and retained knowledge. A total of 207 participants completed both questionnaires. Ten to 12 months after attendance, most participants reported increased confidence undertaking common surgical and clinical tasks; they felt both motivated and able to use their newly acquired knowledge and skills in their workplace, with some peer-to-peer skills transfer opportunities. Many reported high levels of employer engagement resulting in improvements in the workplace, including equipment investment. Evidence for sustained improvements in working practices were noted in four key areas: surgical practices, use of perioperative analgesia, use of perioperative antibiotics, and post-operative wound management. Average knowledge scores in four areas (surgical skills, peri-operative analgesia, post-operative antibiotics and post-operative care) increased significantly 10–12 months after the training programme as compared to before, after accounting for other participants’ characteristics. These findings provide evidence for sustained improvements in workplace practices and patient care after attending a skills-based training opportunity, with a concomitant positive impact on standards of animal welfare. Furthermore, it may inform the development and implementation of future, educational, outcomes-focused training initiatives.

## Introduction

The effectiveness of veterinarians’ abilities to oversee and advocate high standards of animal welfare is strongly underpinned by access to high-quality, educational training opportunities that are relevant to both their professional roles and their individual requirements. The goal of any continuing professional development (CPD) programme is in effecting long-term outcomes evidenced by sustained, behavioural change in the workplace; by inference, this results in improvements in patient welfare and clinical outcomes, which is the key consideration for veterinary professionals. CPD activities that are interactive, provide skills-practice, and focus on outcomes, are more effective at improving practices, and patient health and welfare outcomes compared to isolated activities undertaken outwith defined structured learning (Wallace & May 2016). However, developing formal assessment protocols for CPD programmes that are reliable and sustainable, however, is challenging (Gates *et al.* 2021), not least because there is currently no formally agreed definition in the literature for what constitutes ‘effective CPD’ (Schostak *et al.* 2010). Evaluations can focus on various types of outcomes and frameworks have been devised to assist in this process (Miller 1990; Guskey & Sparks 2000; Moore *et al.* 2004). Whilst it may be relatively straightforward to evaluate the immediate impact of CPD programmes on knowledge, attitude, and skills attainment before and after a training opportunity, demonstrating longer-term outputs can be more challenging.

India has the fastest growing major economy globally and is forecast to become the world’s third-biggest economy in a few years’ time (Banerji 2024; Oi 2024). Like other countries, its veterinary industry must constantly evolve to meet and overcome diverse challenges; one such challenge is developing and delivering effective solutions for managing the country’s large, free-roaming dog population alongside the delivery of rabies control strategies. Veterinarians must be provided with the knowledge and skills to undertake spay neuter surgeries to a high standard of patient care, and to conduct high-welfare, dog population management (DPM) programmes, through the provision of effective, accessible educational training opportunities. These skills are applicable to veterinarians working in multiple different sectors including government or private hospitals, charities or academia. The international veterinary charity, Worldwide Veterinary Service (WVS), has been providing residential 12-day surgical training programmes at its international training centres (ITCs) in India since 2010, with the objective of equipping national veterinarians with the surgical skills and other expertise required for the safe neutering of free-roaming dogs. The programme focuses on providing ‘hands-on’ training to enable the improvement of practical skills in a supportive and non-pressurised environment, closely supervised by nationally trained, Indian veterinary staff. In addition, the skills-focused training is augmented by didactic, lecture-based teaching covering a broad range of surgically related topics, such as animal welfare, multimodal analgesia and the responsible use of antibiotics. A recent paper described the immediate, positive impact of the training programme on Indian veterinarians’ knowledge and confidence through a pre- and post-educational programme assessment (Rayner *et al.* 2023). However, the longer-term impacts of such a skills-focused training programme in India is not known; critical evaluation is needed, not only to facilitate improvements in future content and delivery using an evidence-informed approach, but also in the provision of information to the wider community, such as local governments and NGOs, on the effectiveness of such approaches.

Using structured questionnaires, pre- and around 12 months post-training, the aim of this study was to evaluate the longer-term outcomes of attending a canine surgical-neutering training programme on participants’ knowledge, attitudes and practices, and to evaluate any sustained effect on practices which improve animal welfare.

## Materials and methods

### Ethical considerations

This project was approved by the University of Edinburgh Royal (Dick) School of Veterinary Studies Human Subject Ethical Review Committee in April 2021 (HERC_686_21). The WVS surgery programme was approved by the Animal Welfare Board of India (AWBI), Government of India.

### The educational training programme

The structure of the training programme has been described previously (Rayner *et al.* 2023). Briefly, the study was conducted at two WVS international training centres (ITCs) located in the Indian states of Tamil Nadu and Goa; these were established by the WVS charity in 2010 and 2016, respectively. They provide practical, surgical training in humane DPM to national and international veterinary professionals through regular, 12-day, training programmes taught in English. The programme focuses primarily on providing practical experience in spay neuter surgery overseen by experienced veterinary staff; lectures, practical demonstrations and discussion opportunities are also provided for, covering several key topics (anaesthesia, analgesia, responsible use of antibiotics, fluid therapy, trauma wound management, DPM, animal welfare and rabies).

### Questionnaires

Pre- and post-training questionnaires were compiled which were designed to explore the effect of the educational training programme on participants’ knowledge, attitudes, and practices both during the programme induction (day 1) and 10–12 months after completion (see Supplementary material). At the beginning of the pre-training questionnaire, consent to use the data anonymously for the purpose of the study was requested from individual participants. Participants used a unique identification code for subsequent survey responses. Demographic questions comprised age, gender, professional status and employment sector. A section on working practices explored participants’ previous knowledge of, and practices associated with, undertaking spay neuter surgery; a proportion of these questions were used to assess knowledge. Where participants had not previously undertaken spay neuter surgery, they were asked to complete the questions based on their current knowledge and understanding. This covered areas such as surgical procedures, use of perioperative analgesia and antibiotics, and post-operative wound care. A link to a follow-up questionnaire, alongside their individual user ID for use during completion, was emailed to the participants between 10 to 12 months after completion of the training. The first section comprised a series of questions exploring their professional status since attending the programme; this included employment status, work sector and type, whether they had undertaken clinical work, and specifically, spay neuter surgeries. The second section explored participants’ attitudes regarding their motivational drive to put into practice their newly acquire skills, whether this had been achieved and the barriers which had prevented this. Their attitudes towards their own confidence in undertaking specific, common, clinical and surgical tasks since the training event, was also addressed, as well as their overall confidence as a veterinary professional. A section on working practices identical to the pre-training questionnaire was included to enable a direct comparison with pre-training status; as before, where participants had not undertaken spay neuter surgery since the training programme, they were asked to complete the questions based on their current knowledge and understanding. The final section explored potential impacts of training attendance on working practices including employer engagement, changes to equipment, policies and procedures, and transfer of knowledge and skills to peers. The questionnaires were piloted initially with WVS staff members, and a small number of participants and the results evaluated. Feedback was obtained regarding factors such as clarity and ease of use to improve wording and content. The amended questionnaires were then used in the final study.

The pre-training questionnaire was administered face-to-face using a tablet. Questions were pre-programmed into a mobile device app designed by the WVS charity for the purpose of offline data collection in field conditions (Gibson *et al.* 2018), and this app was used to input the answers at the time of assessment. The data were then synchronised to a secure cloud-based server for review and analysis through a secure website login. The post-training questionnaire was administered online, hosted by the ‘Jisc Online Survey system’ where data were uploaded to a secure server to enable analysis through a secure website login.

### Participant and data inclusion criteria

Indian national participants who attended and completed the canine surgical neutering training programme at either of the two ITCs in India and who provided their consent, were included in the study. Data were included from Indian national participants who completed both the pre-and post-training assessments. Incomplete assessments were excluded.

### Scoring system for knowledge-based questions

A total of 12 questions were used to assess participants’ knowledge of surgical topics both before and 10–12 months after the training event; these were sub-divided into four categories comprising ‘surgical skills’ (seven questions), ‘peri-operative analgesia’ (three questions), ‘post-operative antibiotics’ (one question) and ‘post-operative care’ (one question). For multiple choice questions requiring a single correct answer (n = 11), a score of 1 was assigned for the correct response and 0 for an incorrect response. For the single question where three correct responses existed, a score of 1 was awarded for each correct response chosen and one mark was deducted for each incorrect response selected.

### Statistical analysis

Data analysis was carried out using the R Statistical programme version 3.6.1(R Core team 2019) within RStudio 2020 v1.3.1073 (R Studio Team 2019). R package ggplot2 (Wickam 2016) and Microsoft Excel® (version 2410). was used to generate graphs. The significance level was set at 0.05. A mixed-effects, multivariable linear regression model was built, using questionnaire overall score as the outcome variable and participant ID as a random effect. The aim of the model was to estimate the difference between pre- and post-training scores, adjusting for other factors which included age, gender, employment sector and professional status. Variable selection was carried out using manual backwards elimination. The final model was selected based on lowest Akaike information criterion (AIC) and simplicity.

## Results

### Demographics

The study ran between February 2022 and September 2023 covering 73 training programmes in total with, on average, five participants per course at the ITC in Goa and 12 participants per course at ITC Tamil Nadu. A total of 668 Indian national participants completed the first questionnaire and 234 participants responded to the online questionnaire 10–12 months later, resulting in a 35% response rate. Of these, 207 sets of data were matched and included in the analysis. Demographic data are summarised in Table 1.

**Table 1.** Demographics of participants (n = 207) in the study of veterinarians in India taking part in a canine surgical neutering training programme

		Pre-training (0 months)	Post-training (10–12 months)	Missing values
Demographic				
		Median (range)		
Age		26.5 years (22–55)	N/A	0
		% (number)	% (number)	
Gender	Male	57.0% (118)		
	Female	42.5% (88)		
	Transgender	0.5% (1)	N/A	0
	Non-binary/non-	0.0% (0)		
	conforming	0.0% (0)		
	I’d rather not say	0.0% (0)		
Professional status	Employed / self-employed	62.8% (130)	81.1% (168)	
	Unemployed	21.3% (44)	3.9% (8)	0
	Student-UG	2.4% (5)	0.0% (0)	
	Student-PG	11.6% (24)	15.0% (31)	
	Other	1.9% (4)	0.0% (0)	
Employment sector	Private practice	51.2% (106)	55.9% (105)	
	Government	22.7% (47)	25.0% (47)	
	Charity/NGO	8.7% (18)	12.8% (24)	19 post-
	University staff	4.3% (9)	2.1% (4)	
	Other	13.1% (27)	4.2% (8)	

Key: PG-post-graduate; UG- undergraduate; NGO-non-governmental organisation

At the time of the programme attendance, the median age of the participants attending the programme was 26.5 years (range 22–55 years). The majority were male (57.0%) and employed or self-employed (62.8%). The most common employment sector was private practice which included 51.2% of all participants, whilst the government was the second largest employer (22.7%). Twelve months after the programme, 81.1% were employed and unemployment had reduced from 21.3 to 3.9%. Similar to before the training programme, private practice (55.9%) and government (25.0%) were the most common employment sectors.

Participants’ experience of performing spay neuter surgeries prior to attending the educational programme is summarised in Table 2.

**Table 2.** Participants’ (n = 207) experience of undertaking spay neuter surgery prior to attending a canine surgical neutering training programme in India

Question	Response option	% participants (number)n = 207	Missing values
Are you currently working as a veterinarian?	Yes	79.2% (164)	0
	No	20.8% (43)	
Is canine spay-neuter surgery currently a routine part of your veterinary duties?	Yes	39.1% (81)	0
	No	60.9% (126)	
When was the last time canine spay-neuter surgery was part of your veterinary duties?	Never	42.5% (88)	
	<12 months	39.6% (82)	
	1–2 years	10.6% (22)	0
	>2–5 years	3.9% (8)	
	>5 years	3.4% (7)	
		Median (range)n = 47	
Estimated number of canine spay neuter surgeries completed in last 12 months		5 (1–80)	35

Just over 79% of participants were currently working as a veterinarian. For most participants (60.9%), spay neuter surgery did not form part of their current veterinary duties and 42.5% had never undertaken this procedure. Out of the 57.5% who had, the majority (39.6%) had done so in the previous 12 months, with the total, estimated median value of surgeries completed being five dogs per participant (range of 1 to 80).

Participants’ clinical and surgical experiences 10–12 months after completing the educational programme are summarised in Table 3.

**Table 3.** Surgical working patterns of participants (n = 187) 10–12 months after attending a canine surgical neutering training programme in India

Question	Response option	% participants (number)n = 207	Missing values
Since attending the surgical training, have you worked as a veterinarian?	Yes	90.3% (187)	0
	No	9.7% (20)	
Of those who have worked as a veterinarian since training (n = 187):			
Number of months worked as a veterinarian		2 (median)	0
		1–11 (range)	
		% (value)	
Has your job involved clinical work?	Yes No	96.8% (180) 3.2% (6)	1
Is canine spay-neuter currently part of your veterinary duties?	Yes	69.6% (130)	0
	No	25.1% (47)	
	Not currently working as a vet	5.3% (10)	
Number of participants who have undertaken canine spay-neuter surgery since the programme*		95.2% (178)	0
		Median (range) n = 174	
Number spay-neuter surgeries undertaken per participant		18 (1 to 2,867)	4

* Data calculated from those participants who stated that they had undertaken 1 or more spay-neuter surgeries since attending the training programme

Since attendance, 90.3% (n = 187) had been employed as a veterinarian with duration of employment ranging from 1 to 11 months (median 2 months). Of these, 96.8% (n = 180) reported they had undertaken clinical work. One or more spay neuter surgeries had been undertaken by 95.2% (n = 178) of participants since attending the training programme (the median number of spay neuter surgeries carried out was 18 and ranged from 1 to 2,867); and spay neuter surgery was currently a part of their routine work for 69.6% (n = 130) of participants.

**Figure 1.**
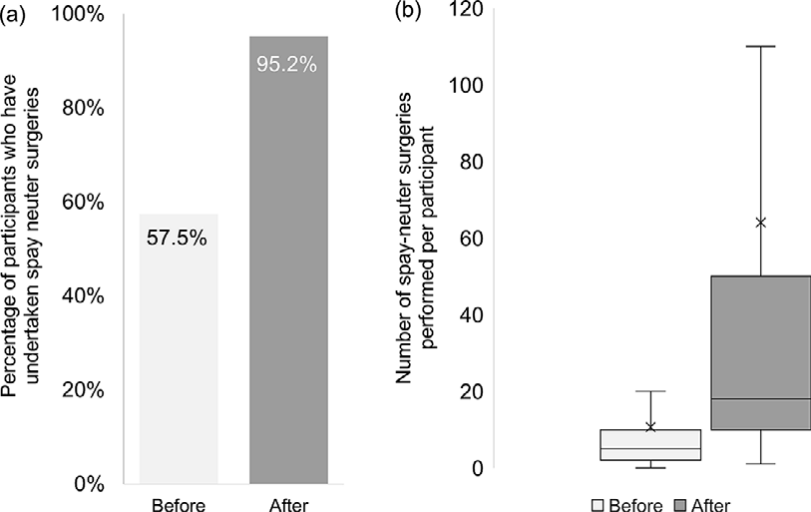
Comparison of the percentage of participants who have (a) undertaken spay neuter surgeries and (b) the number of surgeries performed per individual, both before and 10–12 months after attending a canine surgical neutering training programme in India.

A comparison of the percentage of participants who had undertaken spay neuter surgeries, and the number of surgeries performed per participant, both prior to and after the programme, are shown in Figure 1.

### Knowledge scores

**Figure 2.**
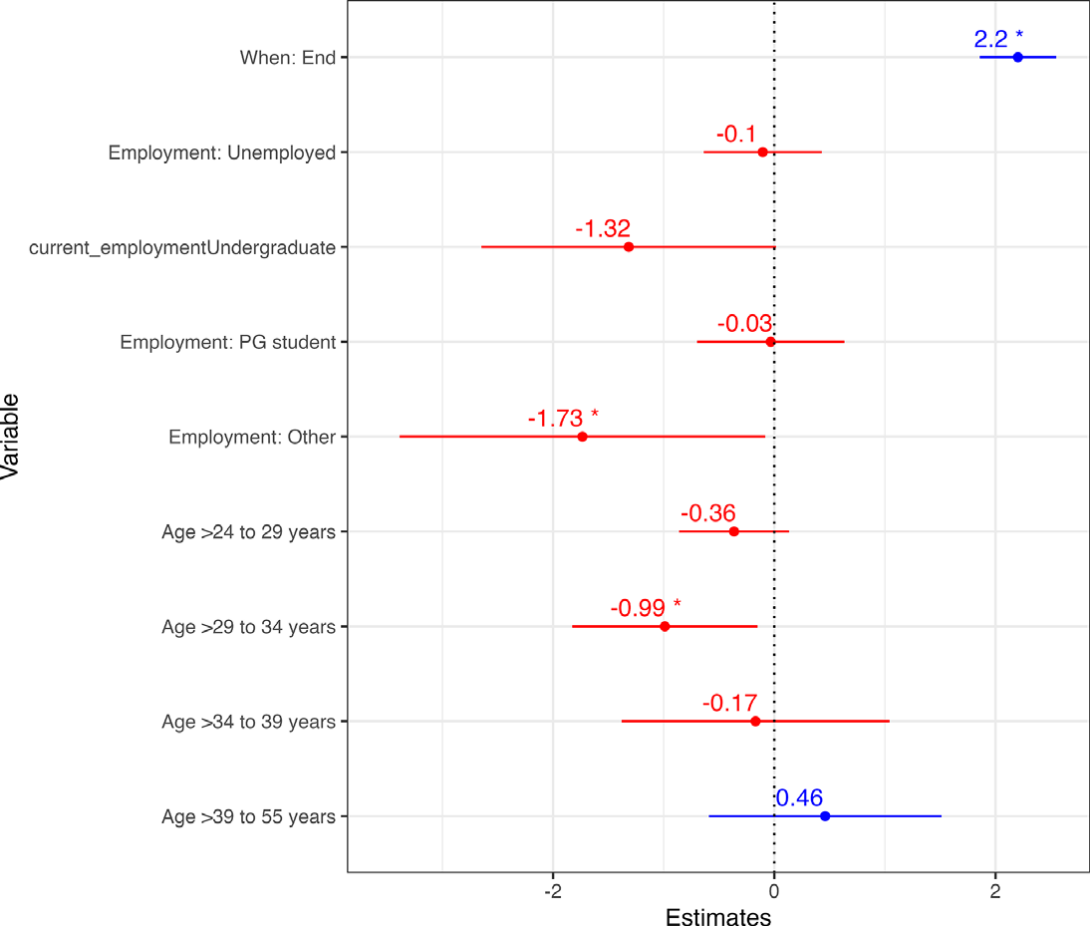
Multivariable linear regression model estimating differences in scores for participants (n = 207) before and 10–12 months after attending a canine surgical neutering training programme in India and adjusting for participants’ characteristics. * Signifies *P* < 0.05.

To evaluate longer-term, knowledge retention, scores from all participants (n = 207) were included in the analyses, regardless of whether or not they had worked as a veterinarian since attending the training programme. The maximum score possible for the 12 questions included in the knowledge assessment was 14. The results are summarised in Figure 2. Regression modelling indicated that average knowledge scores had increased by 2.20 (95% CI 1.86 to 2.55) points 10–12 months after the end of the training programme as compared to before, after accounting for other participants’ characteristics. Those aged > 29 to 34 years and in employment categorised as ‘other’ were associated with significantly lower average scores.

### Self-reported confidence

**Figure 3.**
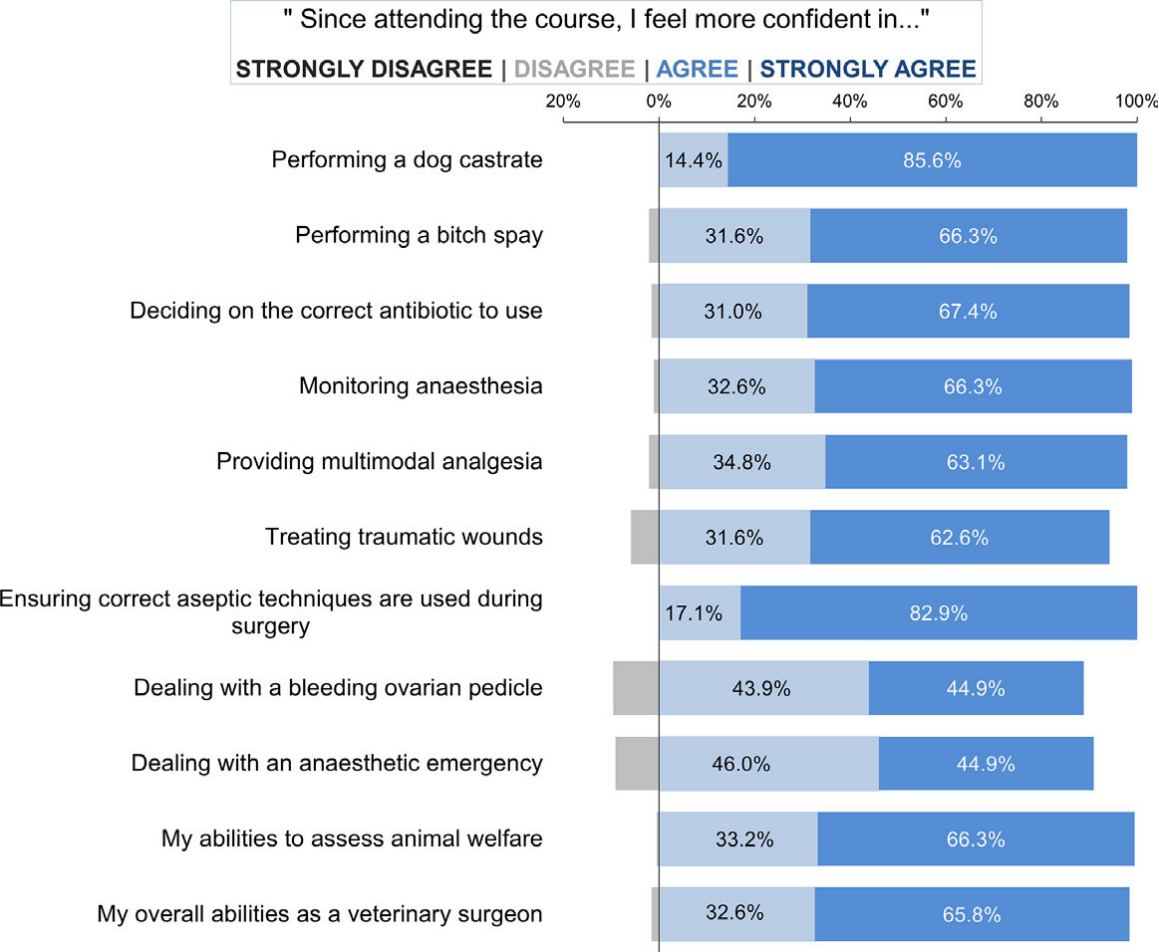
Participants’ (n = 187) self-rated confidence in undertaking surgical and clinical tasks 10–12 months after attending a canine surgical neutering training programme in India.

Attitudes regarding self-confidence in carrying out common surgical and clinical tasks of those participants who had worked as a veterinarian since attending the training opportunity (n = 187) are summarised in Figure 3. There was a high agreement that their confidence had increased in all the tasks since attending the training programme.

### Motivations and opportunities for knowledge/skills usage and transfer

**Figure 4.**
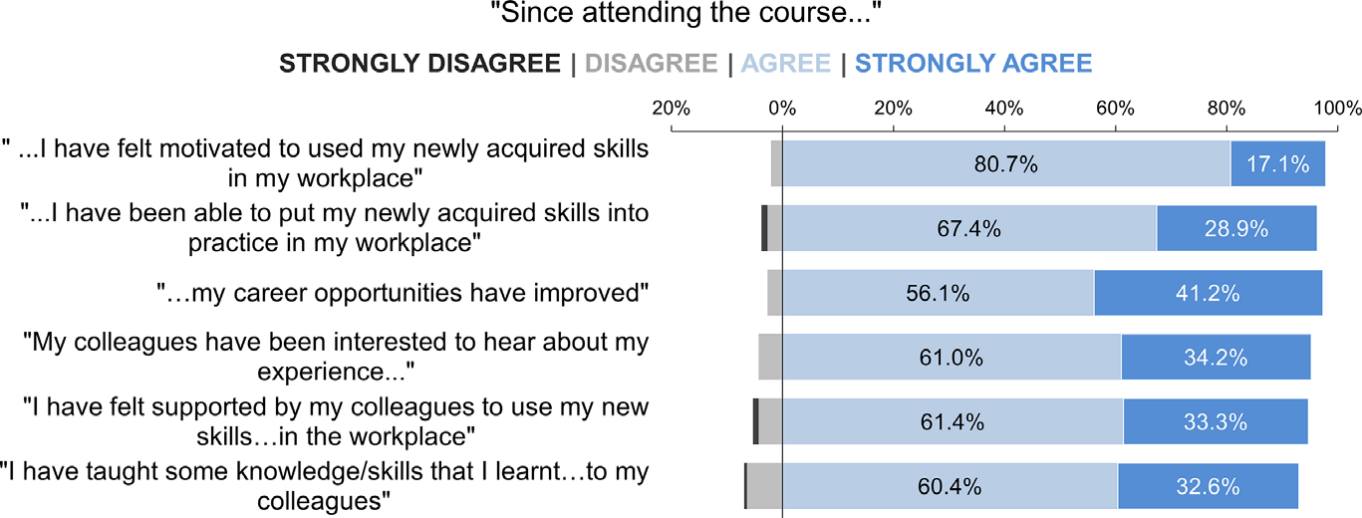
Participants’ (n = 187) motivations and opportunities for skills usage and transfer to workplace colleagues 10–12 months after attending a canine surgical neutering training programme in India.

Participants’ attitudes regarding their motivation to use their knowledge and skills, and the availability of opportunities both to implement these and for transferring these to their colleagues, were explored and the results are summarised in Figure 4. Out of those participants who had worked as a veterinarian since attending the training opportunity (n = 187), 97.8% respondents agreed/strongly agreed that they felt motivated to use their newly acquired skills in their workplace; furthermore, the majority (96.3%) had been able to use these skills at a practical level. Those who were unable to do so were asked to give reasons; these included: “working at a speciality clinic. Procedures like spay and castration are not routine”; “non-clinical workspace”; “I am pursuing my post-graduation in medicine. Unfortunately, I don’t have a good chance of performing OHE and castration”; “no ample opportunity”; and “I have been preparing for NAVLE”. Over 97% agreed/strongly agreed that their career opportunities had been improved through programme attendance. Questions were asked regarding the wider impact of their programme attendance on their workplace colleagues. Participants reported that their colleagues were interested to hear about their experience of the training opportunity (95.2% agreed/strongly agreed) and felt supported by them to use their new skills in the workplace (94.7% agreed/strongly agreed). Furthermore, 93.0% of participants agreed/strongly agreed that they had been able to transfer some of their knowledge and skills through teaching opportunities.

### Impact on workplace practices

The potential impact of the training programme attendance on improving workplace practices, and participants’ experiences discussing and initiating these with their employer, were explored and the findings summarised in Table 4.

**Table 4.** Impact on workplace practices of veterinarians (n = 187) 10–12 months after attending a canine surgical neutering training programme in India

Question	Yes	No
After attending the course, were you able to identify working practices in your workplace which could be improved?	90.4% (169)	9.6% (18)
Of those that answered ‘yes’ (n = 169):		
Did you feel able to approach employer to discuss your suggestions for improving working practices?	92.9% (157)	7.1% (12)
Did your employer allow you to make the changes?	60.9% (103) (some)	10.1% (17)
	29.0% (49) (all)	
Has your workplace invested in any new equipment as a result of you attending the course?	26.0% (44)	74.0% (125)
Has your workplace begun to use any drugs as a result of you attending the course?	32.5% (55)	67.5% (114)

Out of those participants who had worked as a veterinarian since attending the training opportunity (n = 187), 90.4% were able to identify areas for improvement in their workplace based on information they had acquired during the programme, and out of these, 92.9% felt able to approach their employer to discuss them. A high rate of employer compliance followed discussions, with permission for implementation of some (60.9%) or all (29.0%) the proposed suggestions. Resulting from the training programme attendance, just over one-quarter (26.0%) of workplaces invested in new equipment. When asked to provide examples of equipment invested, the following were mentioned: endotracheal tubes, surgical scrub solution, IV catheters, headlamps, weighing machine, radiography equipment, orthopaedic instruments, electro-cautery and general laboratory, surgical and anaesthetic equipment. An autoclave was also repaired. Furthermore, 32.5% of workplaces invested in new clinical drugs: these comprised antibiotics (ampicillin, amoxicillin, ceftriaxone), analgesics (butorphanol, lidocaine, tramadol, buprenorphine), anaesthetic agents (propofol, meloxicam) and an antihistamine (pheniramine).

The following practices relating to four key components of the training programme were investigated further: the use of multi-modal analgesia, the responsible use of antibiotics, surgical practices and post-operative wound management. Participants were asked to base their answers either on their direct experience of undertaking spay neuter or their knowledge and understanding of the process, either before or after attending the training programme (n = 207).

#### Perioperative use of analgesia

Overall, reported standards of practices in the use of peri-operative analgesia were high; these improved after attending the training programme. The prevalence of participants administering pre-emptive analgesia routinely before surgery was 84.1% prior to the training programme, which subsequently increased to 95.7% approximately 12 months later. There was an increase in the administration of routine analgesia to both male and female patients (86.7 to 93.4%). The routine use of post-operative analgesia remained similar (77.3% pre- and 78.7% post-training). The median number of days that post-operative analgesia was routinely provided was three days for both time-points. Results are summarised in Table 5.

**Table 5.** Self-reported routine use of perioperative analgesia during spay-neuter surgery by veterinarians in India (n = 207) before and 10–12 months after attending a canine surgical neutering training programme

Question	Responseoption	% participants (number) n = 207		Missing responses
		Pre-training(0 months)	Post-training(10–12 months)	
Do you give pre-emptive analgesia given routinely before surgery?	Yes	84.1% (174)	95.7% (198)	
	No	9.1% (19)	4.3% (9)	0
	I don’t know	6.8% (14)	N/A	
Choose the option that best describes your practice.	I routinely give analgesia to all female dogs only	2.9% (5)	1.0% (2)	34 pre- 9 post-
	I routinely give analgesia to all male dogs only	0.0% (0)	1.0% (2)	
	I routinely give analgesia to both male and female dogs	86.7% (150)	93.5% (185)	
	It depends on the circumstance	9.8% (17)	4.5% (9)	
	I don’t know	0.6% (1)	N/A	
Do you give analgesia routinely after surgery?	Yes	77.3% (160)	78.7% (163)	
	No	19.3% (40)	21.3% (44)	0
	I don’t know	3.4% (7)	N/A	
		Median (range)	Median (range)	
How many days after surgery is analgesia routinely given?		3 (1–15)	3 (1–20)	0

N/A- ‘I don’t know’ not given as response option for post-course questionnaire.

#### Perioperative use of antibiotics

The routine administration of a pre-operative antibiotic was reported commonly amongst participants, both prior to and after the educational programme (70.0 and 87.9%, respectively); the choice to administer to both male and female dogs also increased from 62.8 to 89.6% after the programme. The most popular route of administration at both time-points was intra-venous, where prevalence increased from 72.9 to 87.4%. Results are summarised in Table 6.

**Table 6.** Self-reported routine use of perioperative antibiotics during spay-neuter surgery by veterinarians in India (n = 207) before and 10–12 months after attending a canine surgical neutering training programme

Question		% participants (number) n = 207		Missing responses
	Response option	Pre-training(0 mths)	Post-training(10–12 mths)	
Do you give antibiotics given routinely before surgery?	Yes	70.0% (145)	87.9% (182)	
	No	27.5% (57)	12.1% (25)	0
	I don’t know	2.5% (5)	N/A	
Choose the option that best describes your practice	I routinely give antibiotics to all female dogs only	1.4% (3)	2.7% (5)	0 pre-
	I routinely give antibiotics to all male dogs only	0.5% (1)	0.0% (0)	
	I routinely give antibiotics to both male and female dogs	62.8% (130)	89.6% (164)	24 post-
	It depends on the circumstance	24.2% (50)	7.7% (14)	
	I don’t know	11.1% (23)	N/A	
Route of administration used	Intravenous Median time*:	72.9% (148) 15 mins	87.4% (160) 15 mins	4 pre- 24 post-
	Intramuscular Median time*:	19.2% (39) 30 mins	8.2% (15) 30 mins	
	Subcutaneous Median time*:	7.4% (15) 10 mins	4.4% (8) 30 mins	
	Other Median time*:	0.5% (1) 1 min	0.0% (0) 0 mins	
Do you give antibiotics routinely after surgery?	Yes	85.8% (175)	55.1% (114)	3 pre- 0 post-
	No	11.3% (23)	44.9% (93)	
	I don’t know	2.9% (6)	N/A	

N/A- ‘I don’t know’ not given as response option for post-course questionnaire. * refers to the time-period between drug administration and start of surgery.

Use of both the intramuscular (19.2% pre- and 8.2% post-training) and subcutaneous (7.4% pre- and 4.4% post-training) routes decreased after the programme; the median time-period from administration to start of surgery remained similar before and after the training programme for the intravenous route (15 min pre- and post-) and intramuscular (30 min pre- and post-); for the subcutaneous route, there was an increase from 10 min pre-training to 30 min post-training. The use of post-operative antibiotics decreased from 85.8 to 55.1% after the programme.

**Figure 5.**
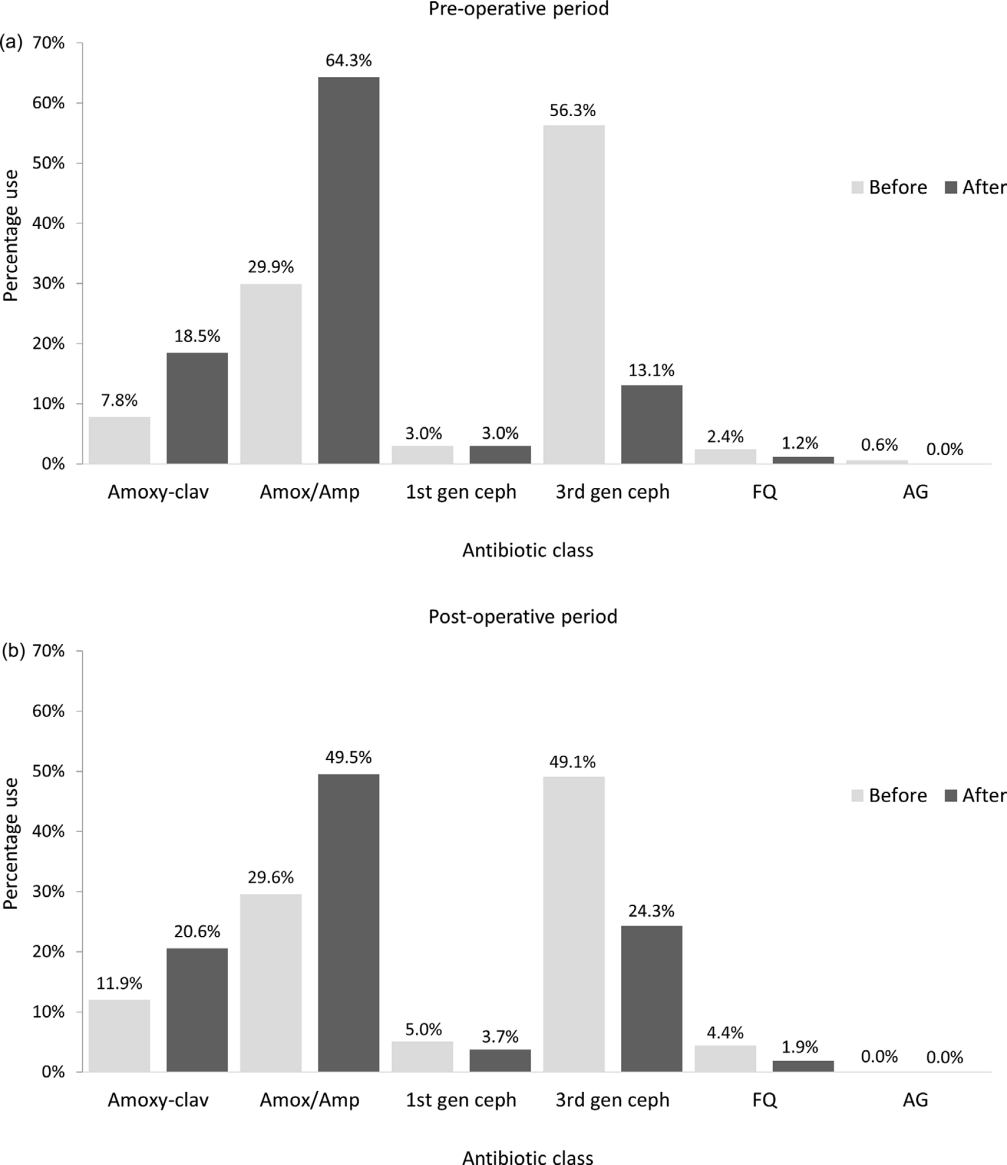
Participants’ (n = 207) choice of antibiotic for using during the (a) pre- or (b) post-operative periods, both before and 10–12 months after attending a canine surgical neutering training programme in India.

Preferences over the class of antibiotics used are summarised in Figure 5. Prior to the training programme, participants favoured the 3^rd^ generation cephalosporins as their drug of choice for both peri- (56.3%) and post-operative (49.1%) use. This was followed by amoxicillin or ampicillin peri-operatively (29.9%), post-operatively (29.6%). After the training programme, there was a decrease in the use of 3^rd^ generation cephalosporins both prior to (13.1%) and post- (24.9%) surgery and a concomitant increase in the use of amoxicillin, ampicillin and amoxicillin-clavulanate. Results from the question “of those who give antibiotics routinely after surgery, how many days are they given” were omitted due to a previously undetected error in the response options which precluded accurate data evaluation.

#### Surgical practices

Participants were asked to choose the response that best matched their routine working practices in the following, pre-operative procedures: endotracheal (ET) intubation, intravenous (IV) canula placement, and IV fluid administration. Results are shown in Table 7. Routine ET intubation of all dogs was performed by 55.7% of participants prior to the training programme and this increased to nearly 80% of participants 10–12 months afterwards. A reduction in the other response options for when ET tubes are used were also noted after the training programme, namely only female dogs (3.6 to 2.4%), only dogs with underlying health concerns (15.5 to 5.9%), only if there is an emergency (6.2 to 3.4%), ET tubes are not available (8.8 to 7.2%) and ET tubes are not used (10.2 to 1.4%). Regarding placing an intravenous cannula routinely during spay neuter surgery, the reported use in all dogs prior to the training programme was already high (86.5%); approximately 12 months after training programme attendance, this had increased to 95.7%. The other response options were low initially and changed only slightly after the training programme; the most notable change was a decrease in the response option “only dogs with an underlying health concern” from 5.0% pre- to 1.3% post-training.

**Table 7.** Self-reported routine use of ET intubation, IV catheter placement and IV fluid administration during spay-neuter surgery by veterinarians in India (n = 207) before and 10–12 months after attending a canine surgical neutering training programme

Question	Response	Percentage of respondents(number)		Missing responses
		Pre-training(0 months)	Post-training (10-12 months)	
Which dogs are routinely intubated during spay-neuter surgery?	All dogs	55.7% (108)	79.7% (165)	13 pre- 0 post-
	Female dogs only	3.6% (7)	2.4% (5)	
	Only dogs with an underlying health concern	15.5% (30)	5.9% (12)	
	Only if there is an emergency	6.2% (12)	3.4% (7)	
	ET tubes are not available	8.8% (17)	7.2% (15)	
	None	10.2% (20)	1.4% (3)	
	I don’t know	0.0% (0)	NA	
Which dogs have an IV cannula placed routinely during spay neuter surgery?	All dogs	86.5% (174)	95.7% (198)	6 pre- 0 post-
	Female dogs only	1.5% (3)	1.0% (2)	
	Only dogs with an underlying health concern	5.0% (10)	1.3% (3)	
	Only if there is an emergency	0.0% (0)	0.5% (1)	
	IV catheters are not available	2.0% (4)	0.5% (1)	
	None	2.5% (5)	1.0% (2)	
	I don’t know	2.5% (5)	NA	
Which dogs have IV fluids given routinely during spay neuter surgery?	All dogs	88.8% (175)	96.6% (200)	10 pre- 0 post-
	Female dogs only	2.0% (4)	0.5% (1)	
	Only dogs with an underlying health concern	3.6% (7)	1.4% (3)	
	Only if there is an emergency	3.6% (7)	0.5% (1)	
	IV fluids are not available	2.0% (4)	0.5% (1)	
	None	0.0% (0)	0.5% (1)	
	I don’t know	0.0% (0)	NA	

N/A- ‘I don’t know’ not given as response option for post-course questionnaire. * refers to the time-period between drug administration and start of surgery.

The absence of placement due to IV catheters not being available decreased from 2.5% (pre-) to 1.0% (post-). Intravenous fluids were administered routinely by nearly 89% of participants before the training programme, increasing to nearly 97% after the event. Whilst 3.6% only gave fluids if there was an underlying health concern prior to training, this subsequently reduced to 1.4% post-training. Preclusion of administration due to IV fluids not being available decreased from 2.0% (pre-) to 0.5% (post-).

#### Post-operative wound management

**Figure 6.**
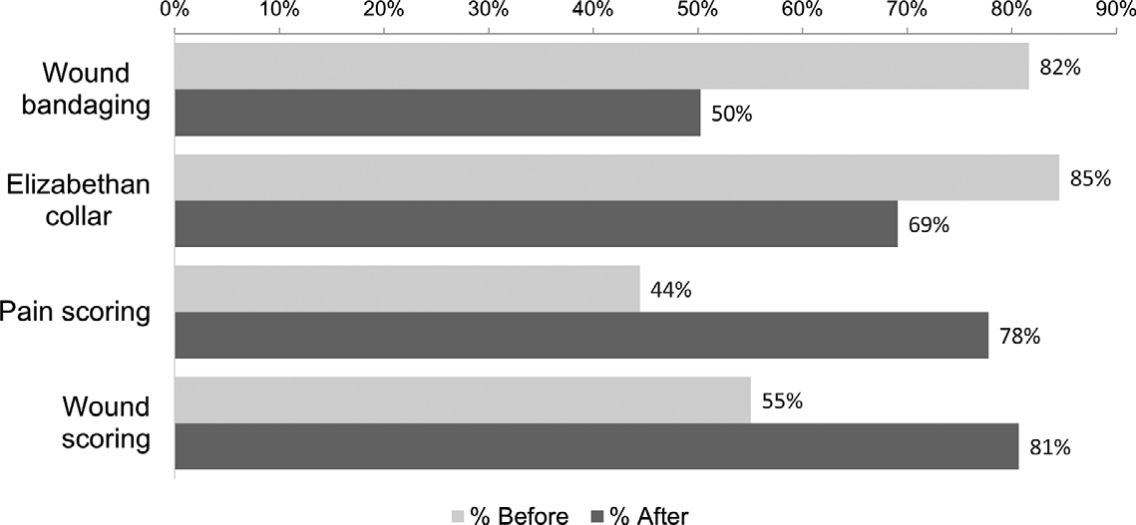
Participants’ (n = 207) post-operative wound practices before and 10–12 months after attending a canine surgical neutering training programme in India.

Participants were asked about their working practices involving post-operative wound management. The responses are illustrated in Figure 6. The prevalence of performing both wound and pain-scoring assessments increased after the surgical training, with an increase from 55 to 81% for wound scoring and an increase from 44 to 78% for pain scoring. A reduction in wound bandaging was noted from 82 to 50%; similarly, there was a modest reduction in the use of an Elizabethan (e-collar) from 85 to 69%.

Lastly, participants were asked to list the areas where they had they had been able to improve working practices within their workplace. Out of the 187 participants who had been employed as a veterinarian since attending the training programme, 169 listed one or more areas: responsible use of antibiotics (n = 123), aseptic practices (n = 119), surgical neutering practices (n = 114), post-operative wound assessment (n = 112), anaesthesia protocols (n = 103), rabies vaccination programmes (n = 94), animal welfare assessment (n = 91), traumatic wound management (n = 81), euthanasia decision-making (n = 73), and rabies diagnosis (n = 62).

## Discussion

The aim of this study was to assess the longer-term impacts on Indian veterinarians who had attended a training programme on canine surgical neutering 10–12 months previously. This follows on from a recently published report demonstrating the immediate improvements in knowledge and attitudes regarding their own confidence amongst a similar cohort attending the same training programme (Rayner *et al.* 2023). Here, we were interested in exploring the sustained effects of the training programme; these included participants’ attitudes regarding their own motivations for using newly acquired knowledge and skills in the workplace setting, their ability to effect changes and how receptive their employers were to this; the type of changes implemented; and opportunities for skills transfer with their colleagues. The period of 10–12 months was chosen as this was deemed adequate time for the development of these described effects within the context of a busy working environment. The practical training programme had a strong ‘hands-on’ ethos which focused specifically on practical sessions to improve skills; this has been shown to address a current, key training need for this specific target audience of Indian veterinarians (Rayner *et al.* 2025). The educational programme was structured with the intention of creating a supportive, non-pressurised environment for participants to develop, practice and consolidate common surgical and clinical skills necessary for conducting canine spay neuter surgeries, as well as other, related, surgical and clinical procedures whilst upholding patient welfare. Therefore, a key programme objective was to improve participants’ confidence in their practical abilities and, thereby, attain the competence necessary to integrate these skills into their future professional activities. It is relatively straightforward to demonstrate short term success of a CPD opportunity using methods such as pre-and post-training assessments; however, to provide evidence of sustained behavioural changes in the workplace and improvements in patient care, an outcome-focused approach is necessitated. Various models for assessing outcomes of CPD activities have been developed; most notably that devised by Moore *et al.* (2009) which has incorporated several other frameworks with the intention of integrating planning and assessment throughout learning activities. Within this overarching assessment, they have incorporated the four levels of competency defined by Miller’s (1990) pyramid, namely: “knows” (declarative knowledge), “knows how” (application of knowledge), “shows how” (competency) and “does” (workplace performance). An alternative, five-level approach has been described by Guskey and Sparks (2000) focusing on CPD outcomes’ assessment within the teaching profession. In addition to capturing aspects of usefulness of the training to participants, it also importantly considers the role of the employer in facilitating the adoption of learning in the workplace. Their five level approach comprises: level 1 - participants’ reactions, such as enjoyment, usefulness and opinions on the learning experience; level 2 - participants’ learning, i.e. knowledge and skills acquisition as defined by the learning objectives; level 3 - organisational (employer) factors to support changes suggested by the participant as a result of training programme attendance; level 4 - use of knowledge and skills, enabling implementation in everyday practice in their workplace; and level 5 - clinical outcomes, i.e. notable improvements in patient care and other areas. These levels provide opportunities for assessment of CPD impact on participants at different stages which can be integrated into an overarching strategy for measuring outcomes (Balmer 2013).

Participants’ feedback on their experiences (aka level 1 of Guskey & Sparks’ approach) and the immediate impacts of any training programme of factors such as knowledge (level 2), are relatively straightforward to evaluate through a pre- and post-training evaluation. These may include knowledge and skills testing, self-evaluation of confidence and general feedback on their experiences (Shea & Shaw 2012; Schnabel *et al.* 2013; Shaw *et al.* 2016; Rayner *et al.* 2020, 2023) and, depending on their format, assess three of the four levels in Miller’s pyramid of competency (‘knows’, ‘knows how’ and ‘shows how’). The fourth level in Miller’s (1990) pyramid of competency (‘does’) requires an assessment focus in the workplace, with a focus on long-term changes in practices and patient outcomes, which was the primary focus of this study. Of particular interest to us was whether participants felt motivated in their workplaces to apply what they had learnt. Numerous reports have shown that simple attendance at a CPD event to be insufficient to motivate behavioural change (Gates *et al.* 2021). Recent research has highlighted the need for skills-based learning opportunities for Indian veterinarians and a shortage of such opportunities (Rayner *et al.* 2025).

The WVS surgical training programmes in India are heavily oversubscribed resulting in high competition for places, including newly graduated, unemployed veterinarians wanting to improve their practical skills and, thereby, chances of employment; it is likely that the attending participants were already motivated to seek out this specific opportunity and, by inference, may be more inclined to implement changes. Indeed, nearly 90% agreed or strongly agreed that they had felt motivated to make improvements in their working practices. However, it is known that intrinsic motivation is also more likely to occur through attainments in knowledge and competency; evidence for this was noted in the significant improvements in knowledge after the training programme compared to before, alongside high, sustained levels of confidence in their abilities. It is suggested that implementing changes in their workplaces and witnessing positive outcomes likely consolidates confidence and an ensuing sense of achievement. Whilst motivation is a driving force required for change, a key factor in the successful implementation of this is the degree of employer compliance (level 3), and this is seldom evaluated (Gates *et al.* 2021). Lack of employer engagement can act as an impediment to veterinarians’ ability to implement their acquired knowledge and skills to improve workplace practices (level 4) and patient care and welfare (level 5), both of which are considered by many as the ‘gold standard’ for evaluating effective outcomes of a training programme attendance. Our study focused on evaluating changes in practices nearly a year after attendance which was considered a sufficient period for these opportunities to occur. We found a high level of support in engagement from employers in both willingness to engage with participants by listening to suggestions for improvements and agreeing to these. It was interesting to note that nearly 98% felt they could approach their employer, with employers supporting implementation of some (63%) or all (30%) of their changes. Whilst the employers included in this study may not be a representative sample of the general veterinary workforce in India, these findings are encouraging; however, it is evident that a degree of resistance from the workforce exists. Of notable consideration is that many of the participants were likely to be junior veterinarians (median age: 26.5 years) and may have only been recently employed in their current positions; as such, this may have been a barrier to the implementation of suggested changes by their employer. Around a quarter of workplaces invested in new equipment and a third in implementing the use of new drugs. Examples of newly purchased equipment comprised: endotracheal tubes for securing the airway during general anaesthesia; surgical scrub solution for facilitating surgical asepsis and reducing the incidence of nosocomial infections; and anaesthetic equipment for improving patient safety and comfort during surgery. Investments in key pieces of equipment are foundational steps in raising standards of clinical care, enhance patient safety and improve their overall welfare experience.

We chose to investigate aspects of the educational programme’s teaching content which we felt were of key importance in conducting canine spay neuter surgery, as well as having wider implications for animal welfare in other surgical and clinical scenarios; these were peri-operative analgesia, the responsible use of antibiotics, surgical practices and post-operative wound management. The aim was to demonstrate, via subjective, self-reporting evaluation, evidence that veterinarians had critically reflected on their current practices and had, subsequently, altered their behaviours accordingly (level 4). The effective use of analgesia throughout the peri-operative period has a profound improvement on the welfare experience of the individual patient undergoing and recovering from a surgical procedure. Our findings showed that the prevalence of routine delivery of peri-operative analgesia improved after the training programme with the largest improvement seen pre-operatively (a rise from 80 to 95% for both male and female dogs). Published findings on the use of pre-operative analgesia in surgical procedures vary amongst veterinary general practitioners globally. In one study, analgesic drugs were routinely administered before and during canine OHE by Brazilian veterinarians (Lorena *et al.* 2014); Swiss veterinarians routinely used pre-operative analgesics for dogs and cats 71–96% of the time for a surgical procedure (Perret-Gentil *et al.* 2014); 98% of Canadian veterinarians reported using analgesics for canine OHE and castration (Reimann *et al.* 2017); and for canine surgeries, 72% of Australian veterinarians reported administering pre-operative analgesia, but only 24% discharging their patients with ongoing analgesia for OHE (Weber *et al.* 2012). It was encouraging to see most veterinarians in our cohort using a pre-emptive approach to pain management, as well as high post-operative pain support, which contributes to a smooth anaesthetic experience, reduced post-operative pain and the promotion of uncomplicated wound healing (Lamont *et al.* 2024).

An important influence on both animal and human welfare is the responsible use of antibiotics. Antimicrobial resistance (AMR) remains a critical, global, health threat and it is estimated that AMR was directly responsible for 1.27 million human deaths in 2019 and contributed to 4.95 million deaths (Murray *et al.* 2022). In India, the use of antibiotics is high, with alarming rates of antibiotic resistance reported (Manesh & Varghese 2021; Vijay et al. 2021b, 2023). Veterinarians are intrinsically positioned in the heart of the human-animal-environment, or One Health, triad and, consequently, hold a position of great responsibility in influencing outcomes. Therefore, they must lead the way in upholding and promoting antimicrobial stewardship (Caneschi *et al.* 2023). There is an urgent need for improved awareness of AMR amongst veterinarians globally; in India, specifically, recent studies have highlighted the focus for clearer prescribing guidance and legislations around antibiotic use by policy-makers (Rao *et al.* 2015; Kumar *et al.* 2019; Mutua *et al.* 2020; Vijay *et al.* 2021a; Eltholth *et al.* 2022). The judicious use of a single, intravenous, pre-operative, narrow spectrum, penicillin-based antibiotic for spay neuter surgeries conducted during the course, with a follow-up dose if surgery time exceeds 90 min, have been considered prudent to mitigate the potential increased risk of surgical site infections whilst participants are learning. However, the course curriculum clearly explains this reasoning to participants as part of a wider, comprehensive teaching session on the responsible use of antibiotics in both routine spay neuter surgeries and other clinical/surgical scenarios in the workplace, in accordance with global guidelines on the responsible use of antibiotics (OIE 2020; WSAVA 2024) and advocate the principles of the British Small Animal Veterinary Association/Small Animal Medicine Society ‘ProtectMe’ guidelines (BSAVA/SAMSoc 2024). Our findings showed that participants’ working practices had changed favourably in several areas after attendance, with a decrease in their routine use of antibiotics post-operatively and an increase in a single dose administered pre-operatively. Furthermore, there was a decrease in the first line use of Category B classes (highest priority critically important antibiotics), such as fluoroquinolones and third-generation cephalosporins (EMA 2020), with a concomitant increase in ampicillin, amoxicillin and amoxy-clavulanate usage. An important consideration when contextualising these findings is that, although the focus of the training programme was on the safe approach to canine spay neuter surgeries in free-roaming dogs, the majority (66%) of participants were employed in private practice after attending, with only 13% employed in a charity/NGO setting. It is possible that participants faced varying degrees of resistance from their employers, particularly those working in private practice, where long-standing practice policies and entrenched attitudes regarding antibiotic use may have prohibited change. By contrast, one could argue that veterinarians overseeing large-scale spay-neuter campaigns to manage dog populations may have more influence on antibiotic protocols. Whilst these findings, overall, are encouraging, further research is necessary to understand current attitudes towards, and barriers preventing, change. Additionally, from a broader perspective, whilst educational initiatives are needed, they must form part of a higher-level, structured and multi-faceted approach to understand the factors influencing the appropriate use of antibiotics (Fazaludeen Koya *et al.* 2022) which can then inform regulatory changes, and support initiatives such as antibiotic stewardship programmes and awareness campaigns.

The most notable improvement in the participants’ surgical practices was the routine use of an ET tube to secure patients’ airways during surgery (increasing from 56 to 80%). Patient safety is improved multi-fold through the provision of a patent airway, facilitation of mechanical ventilation and protection of lower airways from aspiration of fluids (Mosely 2024). The routine use of IV cannula placement and the administration of IV fluids also increased post-attendance. Improvements were also noted in the management of the surgical wound healing with, for example, fewer veterinarians bandaging the wound, and a greater uptake of routine patient pain and wound scoring. All these contribute to improved standards of care during surgical neutering and recovery.

A component of an outcomes-based evaluation of a learning opportunity may also include the knowledge and skills transfer to workplace peers. Indeed, there was a high degree of such sharing of their experiences, with their colleagues reportedly interested and supportive. Peer-to-peer learning has been shown to be effective within the context of undergraduate learning (McMichael *et al.* 2021; Ormandy 2021; Kelly *et al.* 2022) and whilst studies are lacking in the effectiveness of this more *ad hoc* approach to learning in the workplace setting, it is reasonable to assume that this is beneficial, especially when receptivity is high amongst colleagues. This sharing of information may show a degree of similarity with the more formally assembled ‘communities of practice’ (CoP) which is a system of social learning where individuals come together to share knowledge and collaborate around a common theme. The type and extent of peer-to-peer learning was not examined in this study, and whilst it is known that Indian veterinarians communicate and share information regularly through social media (Rayner *et al.* 2025), knowledge sharing may be occurring outwith the workplace via these types of platforms.

The authors acknowledge that there were limitations in the methodology. Firstly, logistical restrictions prevented the inclusion of a comparative control group of veterinarians who had not attended the training programme. Consequently, improvements in variables such as knowledge assessed by the pre- and post-programme assessment may have been influenced by other learning opportunities, such as attendance at other CPD opportunities or peer-to-peer interactions. We also acknowledge that the data were reliant upon participant self-reporting. This approach has the potential to introduce bias, such as social desirability bias, and may have influenced response choices to ones that were predicted to appear more ‘favourable’ by the participant. However, as the follow-up questionnaire was completed online and anonymity was maintained, it was predicted that this likelihood was reduced. The response rate for the second questionnaire was low (35%) and is in accordance with other published data (Moore *et al.* 2004; Wu *et al.* 2022). It is possible that the data may be skewed to include a disproportionately larger number of more motivated respondents, who may be more willing to implement changes. Furthermore, the younger median age (26.5 years) of participants may also be associated with this, as well as other unknown characteristics. Lastly, participants who did not have practical experience of any of the scenarios explored in the pre-training questionnaire were asked to respond from a theoretical perspective based upon their current knowledge and understanding; this may have resulted in some differences in responses compared to their more experienced counterparts. Despite these limitations, the results provide evidence of a sustained improvement in multiple aspects of the working practices of the Indian veterinarian participants, as well as identifying potential opportunities to refine training methods to enhance long-term benefits.

### Animal welfare implications

This study has evaluated the long-term impacts of a skills-focused training opportunity delivered to veterinarians in India; the overarching outcome being to ensure competency in performing their roles to the best of their ability and upholding the welfare standards of animals in their care. We have demonstrated evidence for improvements in long-term knowledge retention and in self-reported practices which will promote enhanced welfare of dogs, and potentially other species, under their care. Within the context of canine surgical neutering of free-roaming dogs, specifically, there are multiple opportunities for animal welfare to be compromised from catching through to release (Bacon *et al.* 2017) and a recent welfare assessment highlighted such parameters that are directly related to the quality of veterinary care, such as analgesia, aseptic technique, surgeon’s experience, length of surgery and dedicated anaesthetic monitoring (Bacon *et al.* 2019). With the current need for relevant CPD opportunities, especially skill-based training, demonstrating their effectiveness provides assurance for positive impacts in patient care and welfare. Overall, these findings will contribute to the current knowledge gap in the literature regarding the long-term effects of this type of training and can help inform the development and implementation of future, educational initiatives.

## Supporting information

Rayner et al. supplementary material Rayner et al. supplementary material

## Supplementary material

The supplementary material for this article can be found at http://doi.org/10.1017/awf.2025.30.

## References

[r1] Bacon H, Vancia V, Walters H and Waran N 2017 Canine trap-neuter-return: a critical review of potential welfare issues. Animal Welfare 26(3): 281–292. 10.7120/09627286.26.3.281

[r2] Bacon H, Walters H, Vancia V, Connelly L and Waran N 2019 Development of a robust canine welfare assessment protocol for use in dog (*Canis familiaris*) Catch-Neuter-Return (CNR) programmes. Animals: An Open Access Journal from MDPI 9(8): 564. 10.3390/ani9080564 31426358 PMC6721185

[r3] Balmer JT 2013 The transformation of continuing medical education (CME) in the United States. Advances in Medical Education and Practice 4: 171–182. 10.2147/AMEP.S35087 24101887 PMC3791543

[r4] Banerji S 2024 *India’s Economy to Remain Strong Despite Subdued Global Growth.* https://www.worldbank.org/en/news/press-release/2024/09/03/india-s-economy-to-remain-strong-despite-subdued-global-growth (accessed 10 March 2024).

[r5] BSAVA/SAMSoc 2024 *BSAVA/SAMSoc Guide to Responsible Use of Antibiotics: PROTECT ME (2024).* British Small Animal Veterinary Association. https://www.bsavalibrary.com/content/chapter/10.22233/9781913859312.chap4_1#supplementary_data (accessed 10 February 2024).

[r6] Caneschi A, Bardhi A, Barbarossa A and Zaghini A 2023 The use of antibiotics and antimicrobial resistance in veterinary medicine, a complex phenomenon: A narrative review. Antibiotics 12(3): 487. 10.3390/antibiotics12030487 36978354 PMC10044628

[r7] Eltholth M, Govindaraj G, Das B, Shanabhoga MB, Swamy HM, Thomas A, Cole J, Shome BR, Holmes MA and Moran D 2022 Factors influencing antibiotic prescribing behavior and understanding of antimicrobial resistance among veterinarians in Assam, India. Frontiers in Veterinary Science 9. 10.3389/FVETS.2022.864813 PMC9087579 35558894

[r8] EMA 2020 Categorisation of antibiotics used in animals promotes responsible use to protect public and animal health. European Medicines Agency (EMA). https://www.ema.europa.eu/en/news/categorisation-antibiotics-used-animals-promotes-responsible-use-protect-public-and-animal-health (accessed 2 October 2024).

[r9] Fazaludeen Koya S, Ganesh S, Selvaraj S, Wirtz VJ, Galea S and Rockers PC 2022 Antibiotic consumption in India: geographical variations and temporal changes between 2011 and 2019. JAC-Antimicrobial Resistance 4(5): dlac112. 10.1093/jacamr/dlac112 PMC9596537 36320447

[r10] Gates MC, McLachlan I, Butler S and Weston JF 2021 Building veterinarians beyond veterinary school: Challenges and opportunities for continuing professional development in veterinary medicine. Journal of Veterinary Medical Education 48(4): 383–400. 10.3138/jvme.2019-0148 34161200

[r11] Gibson AD, Mazeri S, Lohr F, Mayer D, Burdon Bailey JL, Wallace RM, Handel IG, Shervell K, de Bronsvoort BMC, Mellanby RJ and Gamble L 2018 One million dog vaccinations recorded on mHealth innovation used to direct teams in numerous rabies control campaigns. PLoS One 13(7). 10.1371/journal.pone.0200942 PMC6062050 30048469

[r12] Guskey TR and Sparks D 2000 Evaluating Professional Development. Corwin Press: UK. https://uk.sagepub.com/en-gb/eur/evaluating-professional-development/book9582 (accessed 30 September 2024).

[r13] Kelly RM, McCorry MZ, Rackard SM, Doherty ML and Graham H 2022 *Online, student-led, peer-to-peer teaching of clinical skills.* 10.2460/javma.22.10.0455 36434760

[r14] Kumar V, Gupta J and Meena HR 2019 Assessment of awareness about antibiotic resistance and practices followed by veterinarians for judicious prescription of antibiotics: An exploratory study in Eastern Haryana Region of India. Tropical Animal Health and Production 51(3): 677–687. 10.1007/s11250-018-1742-0 30415307

[r15] Lamont L, Grimm K, Robertson S, Love L and Schroeder C 2024 Veterinary Anesthesia and Analgesia: the Sixth Edition of Lumb and Jones*, Sixth Edition.* Wiley Blackwell: London, UK.

[r16] Lorena SE, Luna SP, Lascelles BDX and Corrente JE 2014 Current attitudes regarding the use of perioperative analgesics in dogs and cats by Brazilian veterinarians. Veterinary Anaesthesia and Analgesia 41(1): 82–89. 10.1111/VAA.12104 24344759

[r17] Manesh A and Varghese GM 2021 Rising antimicrobial resistance: an evolving epidemic in a pandemic. The Lancet Microbe 2(9): e419–e420. 10.1016/S2666-5247(21)00173-7 34230918 PMC8248924

[r18] McMichael MA, Ferguson DC, Allender MC, Cope W, Kalantzis M, Haniya S, Searsmith D and Montebello M 2021 Use of a multimodal, peer-to-peer learning management system for introduction of critical clinical thinking to first-year veterinary students. Journal of Veterinary Medical Education 48(2): 170–180. 10.3138/jvme.2019-0029 33433306

[r19] Miller GE 1990 The assessment of clinical skills/competence/performance. Academic Medicine: Journal of the Association of American Medical Colleges 65(9S): S63–S67. 10.1097/00001888-199009000-00045 2400509

[r20] Moore DA, Gilbert RO, Thatcher W, Santos JE and Overton MW 2004 Levels of continuing veterinary medical education program evaluation: Assessing a course on dairy reproductive management. Journal of Veterinary Medical Education 31(2): 146–153. 10.3138/jvme.31.2.146 15181597

[r21] Moore DE, Green JS and Gallis HA 2009 Achieving desired results and improved outcomes: integrating planning and assessment throughout learning activities. The Journal of Continuing Education in the Health Professions 29(1): 1–15. 10.1002/chp.20001 19288562

[r22] Mosely C 2024 Anesthesia equipment. In: Lamont L, Grimm K, Robertson S, Love L, and Schroeder C (eds) Veterinary Anaesthesia and Analgesia*, Sixth Edition.* Wiley: London, UK. https://scholar.google.com/scholar_lookup?doi=10.1002%2F9781119421375.ch3 (accessed 30 September 2024).

[r23] Murray CJL, Ikuta KS, Sharara F, Swetschinski L, Aguilar GR, Gray A, Han C, Bisignano C, Rao P, Wool E, Johnson SC, Browne AJ, Chipeta MG, Fell F, Hackett S, Haines-Woodhouse G, Hamadani BHK, Kumaran EAP, McManigal B, Achalapong S, Agarwal R, Akech S, Albertson S, Amuasi J, Andrews J, Aravkin A, Ashley E, Babin F-X, Bailey F, Baker S, Basnyat B, Bekker A, Bender R, Berkley JA, Bethou A, Bielicki J, Boonkasidecha S, Bukosia J, Carvalheiro C, Castañeda-Orjuela C, Chansamouth V, Chaurasia S, Chiurchiù S, Chowdhury F, Donatien RC, Cook AJ, Cooper B, Cressey TR, Criollo-Mora E, Cunningham M, Darboe S, Day NPJ, Luca MD, Dokova K, Dramowski A, Dunachie SJ, Bich TD, Eckmanns T, Eibach D, Emami A, Feasey N, Fisher-Pearson N, Forrest K, Garcia C, Garrett D, Gastmeier P, Giref AZ, Greer RC, Gupta V, Haller S, Haselbeck A, Hay SI, Holm M, Hopkins S, Hsia Y, Iregbu KC, Jacobs J, Jarovsky D, Javanmardi F, Jenney AWJ, Khorana M, Khusuwan S, Kissoon N, Kobeissi E, Kostyanev T, Krapp F, Krumkamp R, Kumar A, Kyu HH, Lim C, Lim K, Limmathurotsakul D, Loftus MJ, Lunn M, Ma J, Manoharan A, Marks F, May J, Mayxay M, Mturi N, Munera-Huertas T, Musicha P, Musila LA, Mussi-Pinhata MM, Naidu RN, Nakamura T, Nanavati R, Nangia S, Newton P, Ngoun C, Novotney A, Nwakanma D, Obiero CW, Ochoa TJ, Olivas-Martinez A, Olliaro P, Ooko E, Ortiz-Brizuela E, Ounchanum P, Pak GD, Paredes JL, Peleg AY, Perrone C, Phe T, Phommasone K, Plakkal N, Ponce-de-Leon A, Raad M, Ramdin T, Rattanavong S, Riddell A, Roberts T, Robotham JV, Roca A, Rosenthal VD, Rudd KE, Russell N, Sader HS, Saengchan W, Schnall J, Scott JAG, Seekaew S, Sharland M, Shivamallappa M, Sifuentes-Osornio J, Simpson AJ, Steenkeste N, Stewardson AJ, Stoeva T, Tasak N, Thaiprakong A, Thwaites G, Tigoi C, Turner C, Turner P, Doorn HR van, Velaphi S, Vongpradith A, Vongsouvath M, Vu H, Walsh T, Walson JL, Waner S, Wangrangsimakul T, Wannapinij P, Wozniak T, Sharma TEMWY, Yu KC, Zheng P, Sartorius B, Lopez AD, Stergachis A, Moore C, Dolecek C and Naghavi M 2022 Global burden of bacterial antimicrobial resistance in 2019: a systematic analysis. The Lancet 399(10325): 629–655. 10.1016/S0140-6736(21)02724-0 PMC8841637 35065702

[r24] Mutua F, Sharma G, Grace D, Bandyopadhyay S, Shome B and Lindahl J 2020 A review of animal health and drug use practices in India, and their possible link to antimicrobial resistance. Antimicrobial Resistance and Infection Control 9(1). 10.1186/S13756-020-00760-3 PMC7346624 32641109

[r25] Oi M 2024 India economy beats expectations with 8.4% growth. *BBC News.* https://www.bbc.co.uk/news/business-68443347 (accessed 10 March 2024).

[r26] OIE 2020 OIE Standards, Guidelines and Resolutions on Antimicrobial Resistance and the use of antimicrobial agents. World Organisation for Animal Health. chrome-extension://efaidnbmnnnibpcajpcglclefindmkaj/https://www.woah.org/app/uploads/2021/03/book-amr-ang-fnl-lr.pdf (accessed 10 February 2024).

[r27] Ormandy E 2021 Team-based learning within the veterinary curriculum. Developing Academic Practice 2021(April): 1–5. 10.3828/dap.2021.10

[r28] Perret-Gentil F, Doherr MG, Spadavecchia C and Levionnois OL 2014 Attitudes of Swiss veterinarians towards pain and analgesia in dogs and cats. Schweizer Archiv Fur Tierheilkunde 156(3): 111–117. 10.1024/0036-7281/A000560 24568804

[r29] R Core team 2019 *R: A language and environment for statistical computing.* https://www.r-project.org/

[r30] R Studio Team 2019 *R Studio: integrated development for R.* http://www.rstudio.com/

[r31] Rao SVN, Sulaiman VR, Natchimuthu K, Ramkumar S and Sasidhar PVK 2015 Improving the delivery of veterinary services in India. OIE Revue Scientifique et Technique 34(3): 767–777. 10.20506/rst.34.3.2394 27044150

[r32] Rayner EL, Airikkala-Otter I, Bacon HJ, Walters HM, Gamble L and Langford FM 2020 Assessment of an educational intervention on the knowledge and attitudes of indian national veterinarians to animal welfare and euthanasia. Journal of Veterinary Medical Education 47(2): 202–217. 10.3138/JVME.0518-063R/ASSET/IMAGES/SMALL/JVME.0518-063R-FIG3.GIF 31194635

[r33] Rayner EL, Airikkala-Otter I, Mellanby RJ, Gibson AD, Susheelan A, Gamble L and Mazeri S 2023 Assessing the effect of a canine surgical-neutering educational programme on the knowledge and confidence of Indian veterinary participants. Frontiers in Veterinary Science 10. 10.3389/FVETS.2023.942890 PMC10249432 37303735

[r34] Rayner EL, Bastola R, Bedre S, Gibson AD, Gamble L and MacKay JR 2025 ‘No one cares about the animal like me.’ Indian veterinarians’ experiences of improving animal welfare through Continuing Professional Development. Animal Welfare 34: e8. 10.1017/awf.2025.3 39935772 PMC11810509

[r35] Reimann J, Dewey C, Bateman S, Kerr C and Johnson R 2017 Perioperative analgesic use by Ontario veterinarians, 2012. The Canadian Veterinary Journal. La Revue Veterinaire Canadienne 58: 149–156. 28216684 PMC5234314

[r36] Schnabel LV, Maza PS, Williams KM, Irby NL, McDaniel CM and Collins BG 2013 Use of a formal assessment instrument for evaluation of veterinary student surgical skills. Veterinary Surgery: VS 42(4): 488–496. 10.1111/j.1532-950X.2013.12006.x 23581861

[r37] Schostak J, Davis M, Hanson J, Schostak J, Brown T, Driscoll P, Starke I and Jenkins N 2010 ‘Effectiveness of Continuing Professional Development’ project: A summary of findings. Medical Teacher 32(7): 586–592. 10.3109/0142159X.2010.489129 20653382

[r38] Shaw JR, Barley GE, Broadfoot K, Hill AE and Roter DL 2016 Outcomes assessment of on-site communication skills education in a companion animal practice. Journal of the American Veterinary Medical Association 249(4): 419–432. 10.2460/javma.249.4.419 27479287

[r39] Shea A and Shaw S 2012 Evaluation of an educational campaign to increase hand hygiene at a small animal veterinary teaching hospital. Journal of the American Veterinary Medical Association 240(1): 61–64. 10.2460/javma.240.1.61 22171756

[r40] Vijay D, Bedi JS, Dhaka P, Singh R, Singh J, Arora AK and Gill JPS 2021a Knowledge, Attitude, and Practices (KAP) Survey among veterinarians, and risk factors relating to antimicrobial use and treatment failure in dairy herds of India. Antibiotics 10(2): 216. 10.3390/antibiotics10020216 33671483 PMC7926553

[r41] Vijay S, Bansal N, Rao BK, Veeraraghavan B, Rodrigues C, Wattal C, Goyal JP, Tadepalli K, Mathur P, Venkateswaran R, Venkatasubramanian R, Khadanga S, Bhattacharya S, Mukherjee S, Baveja S, Sistla S, Panda S and Walia K 2021b Secondary infections in hospitalized COVID-19 patients: Indian experience. Infection and Drug Resistance 14: 1893–1903. 10.2147/IDR.S299774 34079300 PMC8164345

[r42] Vijay S, Ramasubramanian V, Bansal N, Ohri VC and Walia K 2023 Hospital-based antimicrobial stewardship, India. Bulletin of the World Health Organization 101(1): 20–27A. 10.2471/BLT.22.288797 36593779 PMC9795386

[r43] Wallace S and May SA 2016 Assessing and enhancing quality through outcomes-based continuing professional development (CPD): a review of current practice. Veterinary Record 179(20): 515–520. 10.1136/vr.103862 27856985 PMC5256232

[r44] Weber GH, Morton JM and Keates H 2012 Postoperative pain and perioperative analgesic administration in dogs: practices, attitudes and beliefs of Queensland veterinarians. Australian Veterinary Journal 90(5): 186–193. 10.1111/J.1751-0813.2012.00901.X 22510078

[r45] Wickam H 2016 *ggplot2: Elegant Graphics for Data Analysis.* https://ggplot2-book.org/ (accessed 23 April 2025).

[r46] WSAVA 2024 *Key Documents on Responsible Antimicrobial Use and AMR prevention.* World Small Animal Veterinary Association. chrome-extension://efaidnbmnnnibpcajpcglclefindmkaj/https://wsava.org/wp-content/uploads/2024/08/Key-Documents-on-Responsible-Antimicrobial-Use-and-AMR.pdf (accessed 10 February 2024).

[r47] Wu MJ, Zhao K and Fils-Aime F 2022 Response rates of online surveys in published research: A meta-analysis. Computers in Human Behavior Reports 7. 10.1016/j.chbr.2022.100206

