# Supplementary material for: Evaluation of the longer-term impacts on working practices of veterinarians in India after attending a canine surgical neutering training programme
Source: Anim Welf. 2025 May 30;34:e32. doi: 10.1017/awf.2025.30 (PMC12171784; doi:10.1017/awf.2025.30)
Supplement: Rayner et al. supplementary material [file S0962728625000302sup001.pdf]

Evaluation of the longer-term impacts on working practices of veterinarians in India after attending a canine surgical neutering training programme

Emma L Raynerhttps://orcid.org/0009-0009-6314-3483[1], Anahita Kumar[2], Ilona Airikkala-Otter[3], Stacy Sequeira[2], Richard Mellanby[4], Andrew D Gibson[1,5], Luke Gamble[1], Stella Mazeri[5]

[1] Worldwide Veterinary Service, 4 Castle Street, Cranborne, Dorset, UK

[2] Worldwide Veterinary Service, Hicks International Training Centre Madungo Vaddo, near Assagao Panchayat, Assagao, Bardez, Goa, India

[3] Worldwide Veterinary Service, International Training Centre, Gramya Bhavan, RDO trust Building, Aruvankadu, The Nilgiris 643202, Tamil Nadu, India

[4] Royal (Dick) School of Veterinary Studies, The University of Edinburgh, Edinburgh, UK

[5] The Epidemiology, Economics and Risk Assessment (EERA) Group, The Roslin Institute, Royal (Dick) School of Veterinary Studies, Easter Bush, Midlothian, UK

Author for correspondence: Stella Mazeri, email: smazeri@exseed.ed.ac.uk

Pre-course questionnaire- canine spay neuter surgery course

Thank you for completing this survey. By continuing you confirm that you understand its purpose and can ask questions at any time. You are free to withdraw at any point during the survey up to the point of data analysis. Your data will be used anonymously, and your name will not appear in any report, paper or other document. These data may be used for research purposes, including publication of findings.

Please enter the 6 digit ID-code consisting of TWO CAPITAL letters (your initials) and FOUR numbers (date and month of your birth as ddmm)

1. What is your gender?
    Male
    Female
    Transgender
    Non-binary/Non-conforming
    I'd rather not say

2. What is your age (years)

3. What is your professional status?
    Employed/self employed
    Unemployed
    Undergraduate student
    Graduate studying for PG qualifications
    Other

4. Are you currently working as a vet?
    Yes/No

5. In which veterinary sector do you work?
    Private practice
    Government
    Charity/NGO
    University staff
    Other
    Not currently working

This section explores that practices you use when performing canine spay neuter surgery

6. Is dog spay-neuter currently a routine part of your veterinary duties?
    Yes/No/not currently working

7. When was the last time canine spay-neuter was part of your veterinary duties?
    In the last 12 months
    1-2 years
    >2-5 years
    More than 5 years
    I have never done canine spay/neuter surgeries myself

8. Give an estimate of the number of dogs (male and female) that you have surgically neutered in the last 12 months (if none, add ‘0’)

If you HAVE performed canine spay/neuter surgeries, please describe the surgical practices you used by answering the questions below.

If you HAVE NOT performed spay/neuter surgeries yourself, please answer the questions USING YOUR UNDERSTANDING AND KNOWLEDGE from your training and experience observing surgeries.

9. Which dogs undergoing spay-neuter surgery are routinely intubated throughout the procedure? (select all that apply).
   All dogs
   Females only
   Only dogs with underlying health concerns
   Only if there is an emergency
   Intubation is not available
   I don't know
   None

10. Which dogs undergoing spay-neuter surgery routinely have an IV catheter placed throughout the procedure? (select all that apply).
   All dogs
   Females only
   Only dogs with underlying health concerns
   Only if there is an emergency
   IV catheters are not available
   I don't know
   None

11. Which dogs undergoing spay-neuter surgery are routinely administered IV fluids throughout the procedure? (select all that apply).
   All dogs
   Females only
   Only dogs with underlying health concerns
   Only if there is an emergency
   IV fluids are not available
   I don't know
   None

Please select the type of suture material you use for the following procedures (select all that apply):

12. Ligating an ovarian pedicle
   Catgut
   Absorbable synthetic
   Non-absorbable synthetic

I don't know

13. *Closing the abdominal cavity*
    Catgut
    Absorbable synthetic
    Non-absorbable synthetic
    I don't know

14. *Closing the subcutaneous layer*
    Catgut
    Absorbable synthetic
    Non-absorbable synthetic
    I don't know

15. *Intradermal sutures*
    Catgut
    Absorbable synthetic
    Non-absorbable synthetic
    I don't know

16. *What suture pattern do you use to close the abdominal layer? (Select all that apply)*
    Simple interrupted
    Simple continuous
    Cruciate
    Other
    I don’t know

17. *How many surgeries do you perform with one set of sterile gloves before changing to new gloves?*
    1 surgery
    2 surgeries
    More than 2 surgeries
    Other
    I don't know

18. *How many surgeries do you complete with one set of instruments before washing and autoclaving the set?*
    1 surgery
    2 surgeries
    More than 2 surgeries
    Other
    I don't know

19. *Do you give pre-emptive analgesia routinely before surgery (pre-operative)?*
    Yes/No/I don’t know

20. *If you chose ‘yes’, please choose which option is applicable:*
    I routinely give pre-operative analgesia to all female dogs only
    I routinely give pre-operative analgesia to all male dogs only
    I routinely give pre-operative analgesia to both male and female dogs
    It depends on the circumstance

I don't know

21. *Do you give analgesia routinely after surgery (post-operative)?*
    Yes/No/I don't know

22. If you chose 'yes' how many days after surgery is analgesia administered for?

23. Do you give antibiotics routinely before surgery (pre-operative)?
    Yes/No/I don't know

If 'yes', please answer the following questions. If 'no', please move to question 28.

24. Please choose which option is applicable during spay neuter surgery.
    I routinely give pre-operative antibiotics to all female dogs only
    I routinely give pre-operative antibiotics to all male dogs only
    I routinely give pre-operative antibiotics to both male and female dogs
    It depends on the circumstance
    I don't know

25. Name of antibiotic used.

26. Route of administration used.
    Intravenous
    Intramuscular
    Subcutaneous
    Other

27. How many minutes before the start of surgery are antibiotics administered?

28. *Do you give antibiotics routinely after surgery (post-operatively)?*
    Yes/No/I don't know

29. If yes, please give the name(s) of antibiotic used

30. How many days after surgery are antibiotics routinely given?
    One post-op injection on the day of surgery
    One post-op injection on the day after surgery
    Daily for 3 days
    Daily for 5-7 days
    More than 7 days
    I don't give post-op antibiotics

31. *Please select the practices that you would use for spay-neuter surgeries (you can choose more than one).*
    The surgical wound of female dogs is bandaged
    An e-collar is used
    The patient's pain levels are scored daily.
    The patient's wound is scored daily

Thank you for completing this survey. In 8-10 months' time you will be contacted to complete a follow-up survey online and we would appreciate your cooperation in completing this. Please press 'finish' to submit your answers.

[Note to the reader: the questions in italics indicate those selected to assess knowledge retention].

Post-course questionnaire-canine spay neuter surgery course

Consent: Please read the following consent statement: By giving your consent, you confirm that: You understand the purpose of the survey and can ask questions at any time. You are free to withdraw at any point during the survey up to the point of data analysis. Your data will be used anonymously, and your name will not appear in any report, paper or other document. You understand these data may be used for research purposes including publication of findings.

Please choose one of the following options:

Yes I give my consent/No, I do not give my consent.

If you did not consent to take part, please close your browser window to leave the survey.

……………………………………………….

Section 1: Demographics

Please copy and past the ID code given to you in the email.

1. What is your gender?
   - Male
   - Female
   - Transgender
   - Non-binary/Non-conforming
   - I'd rather not say

2. Since attending the surgical training course, have you worked as a veterinarian?
   - Yes/No

3. How many months have you worked as a veterinarian?

4. Since attending the surgical training, in which veterinary sector have you worked?
   - Private practice
   - Government
   - Charity/NGO
   - University staff
   - Other
   - Not currently working

5. Since attending the surgical training, has your job involved clinical work?
   - Yes/No

6. What is your professional status?
   - Employed/self employed
   - Unemployed
   - Undergraduate student
   - Graduate studying for PG qualifications
   - Other

Section 2: This section explores your attitudes to various factors after attending the course.

Please choose your response (strongly agree/agree/disagree/strongly disagree or 'not applicable') to the following statements:

7. "Since attending the course..."

   I have felt motivated to use my newly acquired skills in my workplace.
   I have been able to put my newly acquired skills into practice in my workplace

8. If you wish, please use this box to comment on the above questions (positive or negative experiences).

9. "Since attending the course, I feel more confident in..."

   Performing a dog castrate
   Performing a bitch spay
   Deciding on the correct antibiotic to use
   Monitoring anaesthesia
   Providing multimodal analgesia
   Treating traumatic wounds
   Ensuring correct aseptic techniques are used during surgery
   Dealing with a bleeding ovarian pedicle
   Dealing with an anaesthetic emergency
   My abilities to assess animal welfare
   My overall abilities as a veterinary surgeon

10. "Since attending the course..."

    My career opportunities have improved

Section 3: Your working practices

This section explores your own practices when performing canine spay neuter surgery

11. Is canine spay-neuter surgery currently part of your veterinary duties?

    Yes/No/I am currently not working as a vet

12. Since attending the surgical training, give an estimate of the number of dogs (male/female) that you have surgically neutered (enter 0 if none).

Please answer the questions below based on either your EXPERIENCE if you HAVE performed spay/neuter surgeries, or your CURRENT KNOWLEDGE/UNDERSTANDING if you HAVEN'T performed spay/neuter surgeries since attending the course.

13. Which dogs undergoing spay-neuter surgery are routinely intubated throughout the procedure? (select all that apply).
    - All dogs
    - Females only
    - Only dogs with underlying health concerns
    - Only if there is an emergency
    - Intubation is not available
    - None

14. Which dogs undergoing spay-neuter surgery routinely have an IV catheter placed throughout the procedure? (select all that apply).
    - All dogs
    - Females only
    - Only dogs with underlying health concerns
    - Only if there is an emergency
    - IV catheters are not available
    - None

15. Which dogs undergoing spay-neuter surgery are routinely administered IV fluids throughout the procedure? (select all that apply).
    - All dogs
    - Females only
    - Only dogs with underlying health concerns
    - Only if there is an emergency
    - IV fluids are not available
    - None

Please select the type of suture material you use for the following procedures (select all that apply):

16. *Ligating an ovarian pedicle*
    - *Catgut*
    - *Absorbable synthetic*
    - *Non-absorbable synthetic*
    - *I don't know*

17. *Closing the abdominal cavity*
    - *Catgut*
    - *Absorbable synthetic*
    - *Non-absorbable synthetic*
    - *I don't know*

18. *Closing the subcutaneous layer*

Catgut
Absorbable synthetic
Non-absorbable synthetic
I don't know

19. *Intradermal sutures*
    Catgut
    Absorbable synthetic
    Non-absorbable synthetic
    I don't know

20. *What suture pattern do you use to close the abdominal layer? (Select all that apply)*
    Simple interrupted
    Simple continuous
    Cruciate
    Other
    I don't know

21. *How many surgeries do you perform with one set of sterile gloves before changing to new gloves?*
    1 surgery
    2 surgeries
    More than 2 surgeries
    Other
    I don't know

22. *How many surgeries do you complete with one set of instruments before washing and autoclaving the set?*
    1 surgery
    2 surgeries
    More than 2 surgeries
    Other
    I don't know

23. *Do you give pre-emptive analgesia routinely before surgery (pre-operative)?*
    Yes/No

24. *If you chose 'yes', please choose which option is applicable:*
    I routinely give pre-operative analgesia to all female dogs only
    I routinely give pre-operative analgesia to all male dogs only
    I routinely give pre-operative analgesia to both male and female dogs
    It depends on the circumstance

25. *Do you give analgesia routinely after surgery (post-operative)?*
    Yes/No

26. If you chose 'yes' how many days after surgery is analgesia administered for?

27. Do you give antibiotics routinely before surgery (pre-operative)?
    Yes/No

If ‘yes’, please answer the following questions. If ‘no’, please move to question 31.

28. Please choose which option is applicable during spay neuter surgery:

    I routinely give pre-operative antibiotics to all female dogs only
    I routinely give pre-operative antibiotics to all male dogs only
    I routinely give pre-operative antibiotics to both male and female dogs
    It depends on the circumstance

29. Name of antibiotic used.

30. Route of administration used.
    Intravenous
    Intramuscular
    Subcutaneous
    Other

31. How many minutes before the start of surgery are antibiotics administered?

32. *Do you give antibiotics routinely after surgery (post-operatively)?*
    Yes/No/I don’t know

33. If yes, please give the name(s) of antibiotic used

34. How many days after surgery are antibiotics routinely given? (If you do not give antibiotics, please leave blank)

35. *Please select the practices that you use for spay-neuter surgeries (you can choose more than one).*
    The surgical wound of female dogs is bandaged
    An e-collar is used
    The patient's pain levels are scored daily.
    The patient's wound is scored daily

Section 5: Your workplace peers

This section is about your workplace colleagues.

If you have worked as a vet since attending the course, please answer the following questions. If you have not worked as a vet since attending the course, please move to the end of the survey.

36. Please choose your response (strongly agree / agree / disagree / strongly disagree) to the following statements regarding your colleagues' responses to your attendance on the surgical training programme. If you have not worked as a vet since attending the course, please move to the end of the survey.

"My colleagues have been interested to hear my experience of the WVS surgical training."
"I have felt supported by my colleagues to use my new skills from the training in the workplace."
"I have taught some of the knowledge and/or skills from the training to my colleagues."

Section 6: Your workplace
This sections focuses on how attending the WVS surgical training may have impacted on your workplace

37. After attending the course, were you able to identify working practices in your workplace which could be improved?
    Yes/No/

38. Did you feel able to approach your employer to discuss your suggestions for improving working practices?
    Yes/No

39. Did you feel able to approach your employer to discuss your suggestions for improving working practices?
    Yes/No

40. Did your employer allow you to make the changes?
    Yes-all of the changes
    Yes-some of the changes
    No-none of the changes

41. Which area(s) have you improved working practices in your workplace (you may select more than one option).

    Surgical neutering techniques

    Aseptic practices

    Responsible use of antibiotics

    Anaesthesia protocols

    Post-operative would assessment

    Traumatic wound management

    Animal welfare assessment

    Euthanasia decision making

    Rabies vaccination programs

    Rabies diagnosis

    Other

42. Has your workplace invested in any new equipment as a result of you attending the course?

    Yes/No

43. Please list the equipment

44. Has your workplace begun to use any new drugs as a result of you attending the course?

    Yes/No

45. Please list the drug name(s)

Thank you for completing this survey. Please provide us with your email address to send your BSAVA free resource link.

[Note to the reader: the questions in italics indicate those selected to assess knowledge retention].
